# Burden and Disease Characteristics of Psoriatic Arthritis at a Tertiary Center

**DOI:** 10.7759/cureus.27359

**Published:** 2022-07-27

**Authors:** Suzan Attar, Amjad M Almanmmas, Shabab M Alamri, Ahmad W Sindi, Majed T Jobah, Marwan A Bader, Abdulrahman K Alghamdi, Majd Z Sahhaf, Turki A AlAmoudi

**Affiliations:** 1 Rheumatology, King Abdulaziz University, Jeddah, SAU; 2 Rheumatology, King Abdulaziz University Hospital, Jeddah, SAU; 3 Medicine, King Abdulaziz University Faculty of Medicine, Jeddah, SAU

**Keywords:** psoriatic arthritis, kauh, arthritis, psoriatic, characteristics, disease, burden

## Abstract

Background: Psoriatic arthritis (PsA) has a detrimental influence on the quality of life (QoL). The goal of this study was to evaluate the QoL of patients with PsA and its determinants at the King Abdulaziz University Hospital in Jeddah, Saudi Arabia.

Methods: A cross-sectional study was conducted on 60 PsA patients. A questionnaire was used to collect data about their demographics. Assessment of health-related quality of life (HRQOL) was done by the Medical Outcomes Study 36-Item Short-Form Health Survey (SF-36). The Functional Assessment of Chronic Illness Therapy (FACIT)-Fatigue and the FACIT-General (FACIT-G) scales were used to assess fatigue. The Bath Ankylosing Spondylitis Disease Activity Index (BASDAI) was used to assess disease activity.

Results: The mean age of the patients was 50.33 ± 11.15 years and 78.3% were females. The mean HRQOL, FACIT-G, FACIT, and BASDAI scores were 59.99 ± 34.67, 28.18 ± 5.95, 20.01 ± 9.68, and 4.05 ± 2.54, respectively. The HRQOL scores were found to have a highly significant negative correlation with both the FACIT and BASDAI scores, as well as the patients' age and BMI. The FACIT-G scores and the BASDAI scores, as well as the BASDAI scores and the FACIT scores, were found to have a strong positive correlation with age and BMI.

Conclusion: PsA has a significant detrimental influence on QoL, with a link between QoL and disease activity and fatigue. A greater understanding of QoL issues will help improve the quality of care.

## Introduction

Psoriatic arthritis (PsA) is a chronic, inflammatory arthropathy associated with psoriasis, probably characterized by bone proliferation and osteolysis, particularly at tendon, ligament, and capsular insertions (entheses) [1,2].

PsA is prevalent among 6-39% of patients with psoriasis and is equally likely to occur in males and females [2]. The annual incidence of PsA for psoriasis patients was reported to be 4.3% in Saudi Arabia [3]. The overall prevalence of PsA in the USA ranges from 101 to 250 per 100,000 people, with an annual incidence reported at 6.6 out of every 100,000 people [3]. The incidence of PsA begins from the age of 30-50 years, but it may begin at a younger age as well [4].

In a study conducted in 2019, the economic burden of PsA was confirmed among UK citizens using 101 patients. The functional status was highly correlated to the direct healthcare cost in this study [5]. Another study conducted in 2017 described how the psychosocial burden affects patients with PsA. Patients suffered from various disorders such as sleep, behavior, stress, and depression [6]. PsA has a great influence on the quality of life (QoL) and it increases the rate of death and morbidity [2].

Patients and society bear a significant economic and QoL burden as a result of PsA. Clinical aspects of PsA, such as concomitant illnesses and disease activity, all lead to a lower physical and psychosocial QoL. The clinical burden of PsA relates to direct medical costs associated with the use of healthcare services [1].

This study aimed to assess the QoL of patients with PsA and its demographic and clinical determinants among patients attending King Abdulaziz University Hospital (KAUH), Jeddah, Saudi Arabia.

## Materials and methods

Study design, setting, and time frame

This cross-sectional study was conducted at KAUH, Jeddah, Saudi Arabia from January to March 2022.

Ethical approval

Ethical approval for the study was obtained from the Unit of Biomedical Ethics, Research Ethics Committee (REC), KAUH, Jeddah, Saudi Arabia (approval number: 218-22; date: 31/5/2022).

Study participants

The inclusion criteria were all patients who attended the follow-up clinic during the study period and agreed to answer the study questionnaire. All patients had a confirmed diagnosis with a consultant, and the total sample of patients who met the inclusion criteria was 60 patients. All PsA patients were included irrespective of the duration of arthritis or the treatment modalities.

Study method

A pre-designed questionnaire was used for data collection that included five sections. The first section included demographic characteristics and smoking status. The second section included an assessment of the health-related quality of life (HRQOL) [7], which is a widely used tool for assessing HRQOL in patients with various rheumatic conditions [8,9].

The third section included the Functional Assessment of Chronic Illness Therapy (FACIT)-Fatigue (FACIT-F) scale. The FACIT-F questionnaire includes 13 items, which are measured on a four-point Likert scale. Thus, the total score ranges from 0 to 52. High scores represent less fatigue. The FACIT-F scale has been previously validated [10,11].

The fourth section included the FACIT-General (FACIT-G) scale (now in version 4). It is a 27-item compilation of general questions divided into four primary QoL domains: physical well-being (PWB), social/family well-being (SWB), emotional well-being (EWB), and functional well-being (FWB). A high score is considered good on FACIT scales. Higher scores on the scales and subscales indicate better QoL. To achieve this, we reverse response scores on negatively phrased questions and then sum item responses. In cases where individual questions are skipped, scores are prorated using the average of the other answers on the scale. The total FACIT-G score is obtained by summing individual subscale scores (PWB + EWB + SWB + FWB). Total scores for the disease, treatment, and condition-specific subscales are obtained by summing all subscale scores (PWB + EWB + SWB + FWB + additional concerns subscale).

The fifth section included the Bath Ankylosing Spondylitis Disease Activity Index (BASDAI) disease activity questionnaire. The BASDAI is an index validated for applying in PsA and contains six questions regarding subjective symptoms during the week prior to answering the questions. Each question is scored on a scale of 0-10. Aside from the last question, 0 indicates none, and 10 indicates very severe. For the last question, 0 is zero hours, 5 is one hour, and 10 is two or more hours. To calculate the BASDAI score, the formula is as follows: BASDAI = ((Q1 + Q2 + Q3 + Q4) + ((Q5 + Q6)/2))/5. The sum score ranges from 0 to 10, and higher values indicate a more active disease [12].

Data analysis

Data were analyzed statistically using SPSS version 26 (IBM Corp., Armonk, NY). To test the relationship between variables, qualitative data were expressed as numbers and percentages. Quantitative data were expressed as mean and standard deviation (mean ± SD). To assess the relationship for quantitative variables, the Mann-Whitney U test, Kruskal-Wallis test, independent sample t-test, and one-way ANOVA were used according to the data normality. Correlation analysis was performed using the Pearson’s and Spearman's tests according to the data normality. A p-value of less than 0.05 was considered statistically significant.

## Results

Table 1 shows that the mean age of the studied patients was 50.33 ± 11.15 years and their mean BMI was 28.72 ± 5.61 kg/m^2^. Of them, 78.3% were females, 90% had a Saudi nationality, 86.7% were married, and 11.7% were current smokers.

**Table 1:** Distribution of study participants according to their demographic characteristics and smoking status (n = 60)

Variable	No. (%)
Age	50.33 ± 11.15
BMI categories	
Normal weight	14 (23.3)
Overweight	25 (41.7)
Obese	21 (35)
BMI	28.72 ± 5.61
Gender	
Female	47 (78.3)
Male	13 (21.7)
Nationality	
Non-Saudi	6 (10)
Saudi	54 (90)
Marital status	
Divorced	3 (5)
Married	52 (86.7)
Single	2 (3.3)
Widow	3 (5)
Smoking status	
Current	7 (11.7)
Never	48 (80)
Past	5 (8.3)

Table 2 shows that the mean HQQOL, FACIT-F, FACIT, and BASDAI scores were 59.99 ± 34.67, 28.18 ± 5.95, 20.01 ± 9.68, and 4.05 ± 2.54, respectively.

**Table 2:** Mean and SD of used scales

Scale	Mean ± SD
Health-related quality of life 36-Item Short Form Survey Instrument (SF-36)	59.99 ± 34.67
Functional Assessment of Chronic Illness Therapy-Fatigue (FACIT-F) scale (version 4)	28.18 ± 5.95
Functional Assessment of Chronic Illness Therapy (FACIT) scale	20.01 ± 9.68
Bath Ankylosing Spondylitis Disease Activity Index (BASDAI) disease activity questionnaire score	4.05 ± 2.54

Table 3 shows that a highly significant negative correlation was found between the HRQOL scores and both the FACIT (r = -0.43, p ≤ 0.001) and BASDAI scores (r = -0.49, p ≤ 0.001), respectively. The same significant negative correlation was found between the HRQOL scores and patients’ age (r = -0.37, p = 0.003) and BMI (r = -0.4, p = 0.001), respectively. While a non-significant negative correlation was found between the HRQOL scores and the FACIT-G scores (r = -0.15, p = 0.254). A significant positive correlation was found between the FACIT-G scores and the BASDAI scores (r = 0.27, p = 0.036). While a non-significant positive correlation was found between the FACIT-G scores and the FACIT scores, age, and BMI of the participants (p ≥ 0.05). A significant positive correlation was found between the BASDAI scores and the FACIT scores and the age and BMI of the participants (p ≤ 0.05).

**Table 3:** Correlation analysis between the scale scores used * Spearman’s correlation; ** Pearson’s correlation.

Variable	HRQOL 36-Item Short Form Survey Instrument (SF-36)	
	r	P-value
Functional Assessment of Chronic Illness Therapy-Fatigue (FACIT-F) scale (version 4)	-0.15*	0.254
Functional Assessment of Chronic Illness Therapy (FACIT) scale	-0.43*	<0.001
Bath Ankylosing Spondylitis Disease Activity Index (BASDAI) disease activity questionnaire score	-0.49*	<0.001
Age	-0.37*	0.003
BMI	-0.4*	0.001
Variable	Functional Assessment of Chronic Illness Therapy-Fatigue (FACIT-F) scale (version 4)	
	r	P-value
Functional Assessment of Chronic Illness Therapy (FACIT) scale	0.23**	0.07
Bath Ankylosing Spondylitis Disease Activity Index (BASDAI) disease activity questionnaire score	0.27**	0.036
Age	0.004*	0.97
BMI	0.15*	0.24
Variable	Bath Ankylosing Spondylitis Disease Activity Index (BASDAI) disease activity questionnaire score	
	r	P-value
Functional Assessment of Chronic Illness Therapy (FACIT) scale	0.75**	<0.001
Age	0.43*	0.001
BMI	0.29*	0.023

Tables 4-6 illustrated that a non-significant relationship was found between the HRQOL, FACIT-F, FACIT, and BASDAI mean scores and all patients’ demographic characteristics and smoking status (p ≥ 0.05).

**Table 4:** Relationship between health-related quality of life 36-Item Short Form Survey Instrument (SF-36) mean scores and participants’ demographic characteristics and smoking status * Mann-Whitney U test; ** Kruskal-Wallis test.

Variable	SF-36 (mean ± SD)	Test	P-value
Gender			
Female	59.47 ± 35.18	3*	0.76
Male	61.88 ± 34.09		
Nationality			
Non-Saudi	67.72 ± 39.83	0.22*	0.239
Saudi	59.13 ± 34.37		
Marital status			
Divorced	59.24 ± 34.53	2**	0.052
Married	32.69 ± 42.35		
Single	92.07 ± 7.68		
Widow	33.14 ± 23.05		
Monthly family income (SR)			
Less than 5000	47.35 ± 37.7	4**	0.981
5000-10,000	61.78 ± 35.49		
10,000-15,000	64.47 ± 33.21		
15,000-20,000	67.23 ± 18.36		
>20,000	60.3 ± 40.85		
Smoking status			
Current	69.42 ± 26.89	2**	0.266
Never	61.14 ± 34.32		
Past	35.78 ± 43.61		

**Table 5:** Relationship between the Functional Assessment of Chronic Illness Therapy-Fatigue (FACIT-F) scale (version 4) mean scores and participants’ demographic characteristics and smoking status * Independent sample t-test; ** one-way ANOVA test.

Variable	FACIT-F scale (version 4)	Test	P-value
Gender			
Female	28.14 ± 5.83	0.08*	0.622
Male	28.3 ± 6.62		
Nationality			
Non-Saudi	29.33 ± 5.2	0.49*	0.675
Saudi	28.05 ± 6.06		
Marital status			
Divorced	28.36 ± 5.96	2.25**	0.114
Married	32.5 ± 2.82		
Single	22 ± 2.17		
Widow	28.18 ± 5.95		
Monthly family income (SR)			
Less than 5000	28.5 ± 4.71	0.41**	0.796
5000-10,000	29.26 ± 5.42		
10,000-15,000	27.15 ± 7.32		
15,000-20,000	26.16 ± 7.47		
>20,000	28.31 ± 5.99		
Smoking status			
Current	29.71 ± 6.66	0.92**	0.404
Never	27.67 ± 5.96		
Past	30.9 ± 4.74		

**Table 6:** Relationship between the Functional Assessment of Chronic Illness Therapy (FACIT) scale mean scores and participants’ demographic characteristics and smoking status * Independent sample t-test; ** one-way ANOVA test.

Variable	FACIT scale	Test	P-value
Gender			
Female	20.08 ± 9.41	1*	0.336
Male	19.76 ± 11.01		
Nationality			
Non-Saudi	13 ± 5.62	1.91*	0.084
Saudi	20.79 ± 9.75		
Marital status			
Divorced	20.45 ± 9.14	1.08**	0.344
Married	20.3 ± 9.1		
Single	20 ± 26.87		
Widow	12 ± 6.92		
Monthly family income (SR)			
Less than 5000	18.18 ± 9.34	2.45**	0.057
5000-10,000	21.15 ± 9.05		
10,000-15,000	22.76 ± 10.94		
15,000-20,000	25.83 ± 8.97		
>20,000	13.45 ± 7.11		
Smoking status			
Current	19 ± 11.66	0.08**	0.923
Never	20.27 ± 9.88		
Past	19 ± 5.24		

The relationship between the mean score of the BASDAI and the demographics of the participants is shown in Table 7. The correlation with gender, nationality, marital status, monthly family income, and smoking status was not significant, and the p-value was ˃ 0.05.

**Table 7:** Relationship between the mean score of the Bath Ankylosing Spondylitis Disease Activity Index (BASDAI) disease activity questionnaire and participants’ demographic characteristics and smoking status * Independent sample t-test; ** one-way ANOVA test.

Variable	BASDAI disease activity questionnaire score	Test	P-value
Gender			
Female	4.14 ± 2.39	0.52*	0.19
Male	3.72 ± 3.11		
Nationality			
Non-Saudi	2.79 ± 1.27	1.28*	0.096
Saudi	4.19 ± 2.61		
Marital status			
Divorced	4.03 ± 2.53	0.18**	0.831
Married	5.07 ± 5.76		
Single	3.7 ± 0.6		
Widow	4.1 ± 2.01		
Monthly family income (SR)			
Less than 5000	3.87 ± 2.15	0.64**	0.63
5000-10,000	4.25 ± 2.28		
10,000-15,000	4.37 ± 3.15		
15,000-20,000	3.04 ± 2.66		
>20,000	4.62 ± 2.94		
Smoking status			
Current	3.63 ± 3.48	0.12**	0.885
Never	4.08 ± 2.53		
Past	4.32 ± 1.33		

## Discussion

PsA is inflammatory arthritis associated with psoriasis [13]. Psoriasis can result in negative impacts on the affected individuals, such as reduced productivity, embarrassment, stress, and lower self-esteem [14]. A previous study from Turkey showed that PsA affected the QoL of patients compared to psoriasis [13]. Psychological and physical disability associated with PsA places a substantial burden on patients because of its diverse clinical spectrum [3].

We investigated the HRQOL, FACIT, and disease activity. The mean score of HRQOL, FACIT-F, FACIT, and BASDAI showed low HRQOL, more fatigue experienced by patients, low QoL, and less active disease. This indicates that although the disease was less active among patients, it negatively affected their HRQOL, fatigue, and QoL [15].

In Lebanon, one study found that 6.4% of PsA patients had a functional disability, and 26% required treatment [15]. In our study, we estimated the mean score of fatigue and not the proportion.

Carneiro et al. conducted a study on PsA patients, and they showed better findings compared to ours, where the mean scores of BASDAI and FACIT-F were 3.59 and 38.3, respectively. The authors demonstrated that fatigue was moderate to intense among less than 25% of patients [4], which was better compared to our findings.

In the current study, HRQOL was negatively and significantly affected by age, BMI, FACIT score, FACIT-F score, and BASDAI score. This means that older patients, patients with higher BMI, and those with more fatigue, less QoL, and more active disease are more prone to have lower HRQOL. Regarding the FACIT-F score of patients, it was significantly and positively associated with the BASDAI score, where those with the more active disease suffered more fatigue. BASDAI scores, in turn, were positively and significantly associated with age, BMI, and FACIT score. Therefore, these items were significantly associated with each other, with the significant association observed regarding age and BMI regarding HRQOL and BASDAI. However, the four investigated items (HRQOL, FACIT-F, FACIT, and BASDAI) were not significantly related to any of the demographics such as gender, nationality, marital status, monthly income, or smoking status.

A previous study showed a significant association between all the domains of SF-36 and FACIT-F [4]. This correlation was not found in the present study, but a negative correlation was found between the SF-36 and the FACIT scale.

A study from Turkey showed a strong positive correlation between disease activity and the score on the health assessment questionnaire (r = 0.6, p ≤ 0.05) [13]. Also, disease activity had a moderate correlation with the FACIT-F score. It should be noted that disease activity was assessed by disease activity in psoriatic arthritis (DAPSA) [13]. Although the previous study used an assessment tool varied from ours to assess the disease severity, there was a similarity between our study and the previous study regarding the correlation between the disease severity and FACIT-F score. However, the correlation was not strong. On the contrary, the association between disease severity and HRQOL in our study was negative; a result that agrees with the Turkish study, where disease severity in PsA patients was related to bad QoL and fatigue level.

A previous study revealed that the QoL among PsA patients was changed due to the physical and psychological impact of the disease [16]. Another study was done to assess inflammatory rheumatic diseases, including PsA [17]. The study showed that all the health concepts of SF-36 were affected by all the investigated rheumatic diseases, including PsA, with physical functioning being the most affected domain due to limitations in functioning [17].

Management of the disease can improve patients' QoL and physical and mental components [18]. It was found that improvements in pain, fatigue, and disability were associated with improvements in physical and mental component scores of the QoL [18].

It was stated that 40% of PsA patients experience more comorbidities, including obesity [3]. In our study, we found that BMI is a predictor of the scores of disease activity and HRQOL, as higher BMI was associated with worse HRQOL and higher disease activity. From this perspective, we can conclude that obesity increases the burden of PsA among patients. In our study, age and BMI were determinants for HRQOL and disease activity. A result that agrees with previous studies [19].

The negative correlation between the mean HRQOL scores and age in the present study was revealed from previous research studies. It was found that young adulthood has been postulated as a critical and sensitive period, in which psoriasis may even have a greater impact on QoL and ultimately on patients’ life course than in older age groups [20,21]. Also, HRQOL, FACIT, FACIT-F, and disease severity affected each other to different degrees as proved by previous studies [20,21], as changes in fatigue were shown to reflect changes in clinical disease activity in PsA. In addition, among PsA patients, the sense of tiredness, loss of energy, and exhaustion not only decreased QoL but also affected academic or work performance, daily activity, exercise level, and social interaction [22].

There was a lack in the number of studies conducted on this subject around the world, and there was no previous Saudi study that assessed this topic before. Moreover, there was no previous study that reported the determinants of QoL, fatigue, and disease activity. These factors clarify the strength of this study. A limitation of the present study was that information about other inﬂammatory conditions and metabolic parameters like diabetes mellitus, hypertension, chronic kidney disease, coronary artery disease, congestive cardiac failure, and cardiovascular accident was not collected, which could inﬂuence the QoL scoring.

## Conclusions

The HRQOL of studied PsA patients had a significant negative correlation with both the FACIT and BASDAI scores, indicating a link between QoL and disease activity and fatigue. The scores of FACIT-G, BASDAI, and FACIT had strong positive correlation with age and BMI. There is a need for assessment of QoL, disease activity, and fatigue in PsA patients for better understanding and intervention to improve the quality of care.
